# Maladaptive personality and the efficiency of response-focused emotion regulation strategies: a study on suppression and acceptance

**DOI:** 10.1186/s40359-025-02873-z

**Published:** 2025-05-31

**Authors:** Elena Trentini, Elise Dan-Glauser

**Affiliations:** Institute of Psychology, University of Lausanne, Géopolis Building, Unil Mouline, Lausanne, 1015 Switzerland

**Keywords:** Maladaptive personality, Emotion regulation difficulties, Emotional responses, Acceptance, Suppression

## Abstract

**Background:**

Personality influences several factors, including cognitive and emotional processes. Previous literature showed that personality affects the individual’s affective system, and that potential emotional difficulties and dysregulations could be associated with maladaptive personality. The Maladaptive Personality Trait Model is a powerful tool for identifying this latter personality type, targeting its psychopathological aspects. The present study first sought to characterize maladaptive personality profiles in terms of potential negative symptoms such as alexithymia, anxiety, depression, and difficulties in emotion regulation. Additionally, we experimentally analyzed the general reactivity to positive and negative emotional stimuli and the efficiency of two response-focused emotion regulation strategies, acceptance and suppression, to understand whether these strategies can be differentially efficient depending on maladaptive personality profiles.

**Methods:**

We defined the personality structure of 103 undergraduate students (Mean_age_: 20.57, SD_age_: 1.90) according to the Maladaptive Personality Trait Model and characterized their emotional features with a survey. In addition, experience, expressivity, and physiological arousal parameters were recorded while participants viewed emotionally charged negative and positive pictures. During this induction, participants either applied acceptance or suppression to regulate emotions, or no specific emotion regulation strategy.

**Results:**

“Resilient” and “Under-controlled” profiles were identified. The Under-controlled group was found to have more emotion regulation difficulties and higher psychopathological risks. Resilient profile reported more resources to regulate emotions and lower scores of depression and anxiety. Regarding emotional induction, both profiles were similarly reactive to both valences in experience and expressivity, but differences were found in physiological parameters. Finally, despite the differential influence of acceptance and suppression on the parameters, they were overall not efficient for any profiles.

**Conclusions:**

This study was one of the first attempt to characterize maladaptive personality in terms of emotion and emotion regulation processes. In addition, it was the first study that investigated the efficiency of suppression and acceptance in relation to maladaptive personality. From a clinical perspective, these results offer a starting point to understand the emotional specificities of maladaptive personality profiles and provide information on how to direct emotion regulation training for this population.

## Introduction

Personality can be broadly defined as a system of recurring and individual thoughts and behaviors that result in specific responses to stimuli [34]. These consistent patterns, which contribute to an individual’s uniqueness, are referred to as *traits* [12] and constitute the core components of personality profiles. Among the various models developed to evaluate personality, the Maladaptive Personality Trait Model (MPTM) is one of the few frameworks that offers a comprehensive taxonomy of the symptomatic aspects of personality [171], thereby facilitating a deeper understanding of psychopathological processes. Personality exerts significant influence on numerous aspects of daily life, such as intelligence and behavior [73], and it has been associated with particular emotional processes [130]. Emotions are also involved in many aspects of a person’s daily life and are elicited by both external and internal stimuli. In some instances, responses to emotional stimuli can lead to negative outcomes, highlighting the importance of efficient emotion regulation (ER) to mitigate such effects. Research has consistently identified emotion dysregulation as a prominent feature of personality disorders, representing a transdiagnostic factor implicated in the persistence of various psychopathological conditions [53, 165]. For example, people high in the Neuroticism trait, a trait included in the Five-Factor Model (FFM), tend to have fewer resources for ER and experience more negative emotions and relationships [127], leading to aversive consequences.

The present study aimed to characterize maladaptive personalities by examining their potential impairments in experiencing and regulating emotions, as well as their possible role as risk factors for the development of other psychological disorders. The latter were assessed with surveys that investigated difficulties in ER, positive and negative affects, alexithymia, and indicative symptoms of depression and anxiety (see Sect. “Emotion symptoms and difficulties measures” for details). In addition, the study assessed reactivity to negative and positive emotions to investigate the influence of personality on the main emotional response parameters (experience, expressivity, and physiological arousal). Finally, the study evaluated the efficiency of two specific ER strategies, acceptance and suppression, to explore whether distinct maladaptive personality profiles may affect their efficiency. With this latter objective, we aimed to determine which strategy might be more appropriate for which maladaptive personality profile.

### Personality and the Maladaptive Personality Trait Model (MPTM)

Among the various models used to assess personality, the FFM stands out as a widely used approach [110]. This model is particularly advantageous as it captures a broad spectrum of personality traits, from non-clinical to clinical manifestations [162], thereby allowing for extensive generalizability across populations. Nonetheless, an alternative personality model, the MPTM, was designed as a clinical tool for assessing the pathological dimensions of personality [162]. The MPTM is integrated within the Alternative Model for Personality Disorders [54], whose broader goal is to characterize personality disorders based on difficulties in personality functioning and maladaptive traits [83]. Like the FFM, the MPTM consists of five main traits: *Negative affectivity, Detachment, Antagonism, Disinhibition* and *Psychoticism*. These traits are generally aligned with the FFM ones [166]. For instance, *Negative affectivity* is aligned with Neuroticism, *Antagonism* with low levels of Agreeableness and *Disinhibition* with low levels of Conscientiousness [148]. However, there are some exceptions. For example, *Psychoticism* showed no strong association with any of the FFM traits [162]. Additionally, while most facets of *Negative Affectivity* align closely with Neuroticism, one of its facets in the MPTM, namely Restricted Affectivity, showed a lower factor loading with Neuroticism [2] than with other traits, suggesting incomplete overlap between the models.

Another important difference between the MPTM and the FFM is the broader psychopathological perspective of the MPTM [122]. Specifically, since the MPTM was developed following the characteristics of personality disorders, it allows a better description of maladaptive aspects of personality that FFM cannot adequately provide [2, 56]. Moreover, MPTM can help delineate the personality profiles associated with pathological disorders and provide suggestions for their subsequent treatment. For example, by analyzing their maladaptive traits and their degree of personality functioning with self-reports, it has been shown that people suffering from polydrug use (consumption of several drugs and addicted to at least one drug, [8]) are more likely to present high maladaptive personality traits than patients with other substance use disorders [117]. In addition, people with high levels of *Negative Affectivity, Disinhibition* and *Psychoticism* are more likely to suffer from internet addiction symptoms [59]. In this context, previous literature [17, 139] investigated personality profiles using the MPTM in clinical and non-clinical populations. Across both groups, some common profiles emerged, such as the Resilient and the Under-controlled profiles. The Resilient profile is characterized by low scores across all traits, whereas the Under-Controlled profile demonstrates high scores in all maladaptive traits except Detachment. Similar results were also retrieved in our last study [156], which investigated healthy undergraduate students.

Hence, and despite its strong clinical foundations, the MPTM taxonomy is also suitable for the study of non-clinical samples. As suggested by Carr and Francis [26], a non-clinical sample may better represent the presence of some etiological factors underlying personality disorders that would be more challenging to find in a clinical sample. This perspective is supported by the decision to validate the Personality Inventory for DSM-5 (PID-5), the primary MPTM assessment tool, by using a non-clinical population [2]. Therefore, findings from the general population can contribute to interpreting clinical data while also offering insights into the pathological personality traits that are present in normative populations. Finally, as suggested by Liu et al. [103], the assessment of maladaptive personality can also be helpful in detecting potential risk factors, especially related to emotion processes and emotion dysregulation, given that these are often associated with MPTM and broader personality disorders.

### Emotion and emotion regulation

Emotions constitute a significant aspect of human life. Despite the lack of consensus on the definition of emotions, they can be characterized as transient events that involve several consequences in various systems of the organism. Prior literature investigated the parameters involved in emotional reaction. An emotional reaction can be detected in the subjective experience [16], in expressivity [49], in physiological arousal [81], or in all of these parameters, as Scherer [143] suggested. Depending on how the emotional events are interpreted, a cascade of emotional and behavioral responses is elicited. For example, a misinterpreted comment about someone’s job could trigger a chain of emotions like anger and frustration that can lead to impulsive actions and possible negative situations. When an individual more frequently evaluates an emotional stimulus as negative rather than positive, there is a higher likelihood of experiencing frequent negative affects, which could lead to a negative mood in response to various situations [161] and, in some cases, to a higher risk of developing depression [140]. At the same time, a lack of awareness of emotional stimuli is related to alexithymia, defined as a difficulty in describing and identifying one’s own feelings, which often results in reduced capacity for ER [29]. This creates a negative loop of negative affects, impairments in understanding emotions, and possible ER difficulties, as suggested by prior literature [25, 119, 137].

ER is defined as the attempt to regulate one’s emotions (automatically or consciously) in order to shape emotional outcomes in one’s favor [66]. This can range from simply decreasing negative emotions to achieving specific goals [50, 86]. In Gross’s model [67], several ER strategies are described and categorized into two main forms, each with a specific time frame for action along the emotion-generative process: a) the antecedent-focused phase, where emotions can be regulated by shaping the stimulus, attention or thought focus; and b) the response-focused phase, where emotional responses are already present and are specifically targeted by the regulation. Previous studies have sought to determine which strategy is more efficient, quantify the cognitive or physical efforts they require, and examine the consequences that follow their implementation (e.g., [4, 38, 48, 68]). Based on these studies’ results, strategies were classified as either adaptive or maladaptive. More specifically, they posit that strategies considered adaptive efficiently reduce negative emotions and help achieve specific goals. In contrast, maladaptive strategies typically fail to modulate emotional responses and increase the risk of psychopathological disorders [3]. However, several studies yielded contrasting results, making it challenging to definitively classify a strategy as adaptive or maladaptive.

### The emotion regulation strategies of acceptance and suppression

To disentangle these conflicting findings across studies, we decided to focus on two specific strategies: acceptance and suppression. Both are response-focused strategies, but acceptance is typically considered adaptive, while suppression is often considered maladaptive.

Acceptance can be defined as the enhanced awareness of the feelings associated with the current situation, coupled with the acceptance of such emotional responses, without any attempt to avoid or control them [38, 157]. The ultimate goal is to change the way a person manages thoughts about emotion responses in a non-judgmental way [157]. Additionally, acceptance is one of the core components of the Acceptance and Commitment Therapy. This treatment is designed to enhance psychological flexibility in order to raise psychological awareness and live the experiences in a more conscious way. Counterintuitively, this approach can lead to less intense emotional experiences and quicker recovery from emotional episodes. Acceptance is recognized as an adaptive strategy because of the cognitive and physical benefits it provides. In a non-clinical population, acceptance has been shown to reduce pain [74] and negative emotions [169]. Furthermore, Dan-Glauser and Gross [38] reported greater emotional expressivity compared to suppression and the unregulated condition during both the viewing of negative and positive stimuli. This result was considered by the authors as an advantage of acceptance, as the increased expressivity may promote better social and adaptive interactions between people. Always within the non-clinical population, and in terms of physiological arousal, acceptance has been shown to reduce physiological reactivity [157] and to result in a smaller increase in electrodermal activity in the five minutes following the viewing of a stressful video [47], while Dixon-Gordon et al. [46] reported increased heart rate variability in Borderline Personality Disorder (BPD) patients when acceptance was applied. A final benefit of acceptance lies in the improvement of several psychological disorders, such as eating disorders or addictions [168]. Despite the many positive outcomes, acceptance also led to potentially more aversive effects. For example, Germain and Kangas [58] observed an increased state-anger during acceptance compared to during reappraisal and suppression after implementing these strategies in a non-clinical population. Similarly, Campbell-Sills et al. [23] found that, compared to suppression during the presentation of a negative video, acceptance initially led to increased negative emotions.

Suppression is related to attempts to control and repress emotional responses provoked by the current situation, avoiding showing any emotional reaction to others [67]. Previous studies have suggested that this strategy requires more cognitive resources, consequently leading to more physiological costs [86, 100, 120]. However, other studies have found benefits in suppression. For example, Kobylińska et al. [87] found that suppression can be effective in reducing the expressivity of negative emotions in healthy populations, and it may thus be more suitable for regulating emotional behavior. Furthermore, as shown in a previous study [155], suppression does not lead to a decrease in positive experiences for a short period of time.

Given the discrepancies between studies in assessing the efficiency of ER strategies, external factors likely influence ER efficiency. One of these moderating factors could be personality [153], and in particular maladaptive personality, which presents higher potential risks of emotion dysregulation. Therefore, deepening the knowledge of the interaction between maladaptive personality and emotions may explain the contrast between previous results and, more importantly, could potentially be pivotal in treating emotion dysregulation in patients with high maladaptive personality traits.

### Maladaptive personality and emotions

Personality is an influential factor in emotions. In the case of maladaptive personality, it may negatively influence general affect levels [127], and lead to possible heightened alexithymia levels [56], as well as depression and anxiety [2, 62]. Furthermore, personality appears to exert an influence on the intensity of some emotional responses, with some contrasting results, particularly in the context of BPD. Previous studies have demonstrated a general hyporeactivity in expressivity [131], and in certain physiological parameters such as the skin conductance level (SCL, [76]) in individuals with BPD. At the experiential level, Linehan [102] reported an overall longer and more intense emotional reaction, regardless of the valence of the stimulus. In contrast to this, another study found no differences over time in the subjective rating of joy [80] in BPD patients. Regarding the maladaptive profiles, Trentini and Dan-Glauser [156] found differential reactivity in emotional responses depending on personality profiles. The Under-controlled profile exhibited an emotional reaction evidenced by variation in respiration rate (RR) for positive and negative images and in Skin Conductance Level (SCL) during negative images, whereas the Resilient group demonstrated modifications in heart rate (HR) and RR for both valences, and in SCL for positive images.

Concerning personality and ER, maladaptive personality has been linked to emotion dysregulation [127], a construct representing a key domain in the development of the MPTM [54]. Emotion dysregulation is characterized by the dysfunctional and inappropriate use of ER strategies [36], a lack of awareness or acceptance of emotions, and difficulty in handling impulsivity [45]. In examining the relationship between maladaptive personality and emotion dysregulation, Rogier et al. [134] found that individuals with impulsivity (a facet of the *Disinhibition* trait) demonstrated reduced capacity to apply reappraisal. Furthermore, suppression was linked to paranoid thoughts and was typically observed in people with alexithymia [27, 95]. Conversely, acceptance was associated with a reduction of these pathological thoughts [14].

A comparison of the efficiencies of ER strategies in maladaptive personality revealed that BPD patients showed a greater decrease in negative emotions during emotional avoidance than during acceptance [27]. Trentini and Dan-Glauser [156] also showed that the dual influence of belonging to the Resilient profile reinforced the efficiency of reappraisal in decreasing negative emotions. Conversely, individuals in the Under-controlled group who implemented the same strategy demonstrated non-significant results. Furthermore, using suppression was not efficient at the experience level for this group, and even counterproductive. This result was comparable to those of Pizarro-Campagna et al. [126], who investigated reappraisal and suppression in young early-stage BPD and healthy participants. The BPD group did not benefit from reappraisal, but, in contrast to Trentini and Dan-Glauser [156], they benefited from the application of suppression. Despite these initial attempts to understand the relationship between maladaptive traits and efficiency of ER, research results in this domain remain scarce and further investigations are required.

### The present study

As described above, ER and personality play a role in the emotional process and can lead to clear differences in emotional responses, as well as in the efficiencies of ER strategies. Our study had four main objectives. The first goal of the study was to obtain profile personality groups according to their maladaptive traits. Given the scarcity of literature on the emotional aspects of certain maladaptive profiles, the second goal was to detect some of the emotional characteristics of such profiles and their potential risk factors in this domain. With this objective, we wished to provide a comprehensive overview of maladaptive profiles in terms of how these groups deal with emotions and the specific difficulties they may encounter. To do this, participants completed a series of questionnaires addressing alexithymia, general positive and negative affects, difficulties in ER, and the presence of depression and anxiety symptoms. The third objective was to examine the major emotional responses (experience, expressivity, and physiological arousal) during the emotional induction of negatively and positively valenced emotions. This was done to test how these measures may react and coherently differ from each other in different maladaptive personality profiles. Finally, our fourth objective was to target ER efficiency with a particular focus on examining the acceptance and suppression implementation effects across different maladaptive personality profiles. More specifically, we wanted to assess whether these two strategies have different efficiencies for different maladaptive profiles, which would go beyond a rough adaptive/maladaptive labeling of these strategies, and which might explain previous discrepant findings. We analyzed the efficiency of acceptance and suppression ER strategies using the Difference Index (DI) methodology, which has previously been employed in other studies [152, 155, 156]. The DI can be interpreted as a measure of strategy efficiency, i.e., the extent to which a particular strategy elicits regulated responses, in comparison to a natural unfolding of emotional outcomes. We considered efficient a strategy that reduces negative emotional responses and an increase of positive ones. Given the originality of the study, our hypotheses were primarily exploratory in nature.

- H1) Regarding personality profiles, we expected that two principal clusters would emerge, analogous to those identified in previous studies [17, 156]: the Resilient and the Under-controlled profile. The Resilient group was expected to have low scores in all maladaptive traits while the Under-controlled group would gather high scores in all maladaptive traits.

For the sake of consistency, the same personality profile names were used in the subsequent hypotheses.

Regarding the characterization of personality profiles for emotion difficulties we expected the following results:

- H2) In accordance with the findings of Garofalo et al. [56], who retrieved a positive correlation between all maladaptive traits and alexithymia, we expected that individuals in the Under-controlled group would display elevated levels of alexithymia. We also expected for this group a higher association with negative affects and lower positive affects for the Resilient people, in accordance with Pollock et al. [127]. Based on Bastiaens et al. [17] and Rossi et al. [139], it was also hypothesized that Under-controlled group would experience more difficulties in regulating emotions than the Resilient group. Finally, we expected that the Under-controlled personality cluster would exhibit higher levels of depression and anxiety than the Resilient cluster, in accordance with Gonçalves et al. [62].

Regarding emotional reactivity in relationship with personality:

- H3) We expected that the Under-controlled group would exhibit heightened reactivity in the domain of experience, whereas we anticipated a diminished intensity of emotional expression in the domain of expressivity. These expectations were informed by the findings of Abdi and Pak [1]. With regard to the physiological parameters, it was anticipated that the Under-controlled individuals would display a higher level of physiological reactivity than those who were in the Resilient group. The only exception would be for the SCL parameter, where we expected lower levels of SCL in the Under-controlled cluster. Indeed, as reported Herpertz et al. [76], SCL levels were lower in antisocial and BPD, and these disorders may be representative of Under-controlled group [139].

Finally, to examine the efficiency of suppression and acceptance according to personality, we focused on the three main emotional responses and had the following expectations:

- H4.1) Regarding experience, we expected that acceptance would be more efficient than suppression for Resilient people. We also expected that suppression would not be efficient in Under-controlled people, as reported by Trentini and Dan-Glauser [156] and Bahlinger et al. [14].
- H4.2) Regarding acceptance in expressivity, we hypothesized that the Resilient group would derive greater benefit from this strategy than the Under-controlled group, particularly toward positive images [38]. Regarding suppression, a previous study in our laboratory [156] did not reveal a statistically significant difference between reappraisal and suppression, regardless of personality. Nevertheless, we wanted to replicate the result, with the expectation that a difference would emerge with regards to acceptance. Specifically, we expected that suppression would lead to a greater reduction in expressivity during negative viewing as compared to acceptance. For positive viewing, we predicted that suppression would not result in an increase in expressivity, in contrast to what would be observed with acceptance.
- H4.3) Despite the lack of an overall significant difference in physiological arousal between acceptance and the unregulated condition reported by Dan-Glauser and Gross [38], it was hypothesized that the addition of personality factor could modulate the results. Specifically, a greater reduction in physiological intensity in the Resilient group compared to the Under-controlled group during negative viewing. Regarding suppression, the findings of Trentini and Dan-Glauser [156] indicated that this strategy did not show significant differences in physiological parameters compared to reappraisal, particularly when personality was taken into account. Nevertheless, in this case, we expected significantly higher physiological levels during suppression than during acceptance, especially for Under-controlled individuals. This because of the high physiological costs that suppression provokes [86, 100], along with the impulsive tendency of Under-controlled people [160] that could reinforce the presence of high physiological activity.

## Methods

### Participants

Of the 152 participants who were initially tested, 103 were retained (64 women and 39 men) after the exclusion of participants whose data were lost due to technical issues. The sample included 40 first-year psychology students and 63 students from other faculties. Participants were between 18 and 32 years old (Mean_age_ = 20.57, SD_age_ = 1.90). The choice of a non-clinical sample allowed a comparison with previous literature results, obtained with similar samples. In addition, evidence suggests that the general population displays a range of both adaptive and maladaptive traits [6, 61, 167]. A-priori power analysis for sample size determination indicated that a total sample size of *N* = 72 was required (power at 0.80 and α = 0.05, [52]) to detect a large effect. From there, we increased the number of participants by 40% to compensate for potential data loss (technical issues or artifacts reduction during the signal processing). The experiment was conducted in French and, therefore, participants were required to be native French speakers or have an excellent knowledge of French. For this experiment we had several exclusion criteria based on prior literature. We asked the interested people not to enlist if they were left-handers [19], pregnant women [60, 113], people who regularly consumed drugs [85] and/or alcohol [124], took regular medication, and if they had mood disorders [82, 107] or a diagnosis of anxiety [10, 31]. Psychology students recruited for the study were rewarded with course credit, while participants from other faculties received 50 CHF.

### Measures

#### Personality measures

The maladaptive personality traits of the participants were assessed using the Personality Inventory for DSM-5 Short Form (PID-5-SF), an abbreviated form of the PID-5, consisting of 100 items [93, 127, 149, 154]. This survey, developed by the DSM-5 Personality and Personality Disorders Workgroup, links personality traits and psychopathological disorders, thereby facilitating a more comprehensive understanding of the latter and providing an alternative taxonomy to former diagnoses [94, 170]. In parallel, it allows a reliable assessment of the level of each of the maladaptive personality traits of the respondent. As participants were French speakers, we used the French validated version of this survey [138]. In our sample, the Cronbach’s alpha coefficient ranged from 0.82 to 0.88 for the five maladaptive traits.

#### Measures of emotion symptoms and difficulties

The initial objective was to more precisely characterize the personality profiles in terms of emotional symptoms and difficulties. To do this, participants were requested to complete a series of self-report questionnaires after the session in the laboratory. Four specific instruments were targeted, all of which presented in their French version.

The initial instrument employed was the Toronto Alexithymia Scale (TAS, 20-items, [13]) in its French version [20]. The TAS is an instrument designed to assess with 20 items 5-point Likert scales the degree of alexithymia present in participants, primarily by evaluating their capacity to describe and identify feelings [96]. It consists of three subscales: difficulty in describing feelings (five items, e.g., “*It is difficult for me to find the right words for my feelings*”); difficulty in identifying feelings, i.e., the struggle to recognize an emotion as it is (seven items, e.g., “*I am often confused about what emotion I am feeling*”); and externally-oriented thinking, which is the tendency of individuals to refrain from introspective reflection on their own feelings (eight items, e.g., “*I prefer to just let things happen rather than to understand why they turned out that way)*. Additionally, a total score was calculated. In our sample, Cronbach’s alpha values for this measurement ranged from 0.55 to 0.76.

Second, the French version of Positive and Negative Affect Schedule (PANAS, 20-items, [18]) was employed to assess how frequently participants felt positive (10 items) and negative (10 items) affects. PANAS instructions can be adapted to reflect state or dispositional affects. In the present study, we tested dispositional affects of participants with the instruction “*In this questionnaire, you’ll find words that express different emotions. Read each word carefully and indicate how frequently you feel this way*”. In our sample, the positive affect scale Cronbach’s alpha was 0.83, while the negative affect scale one was 0.79.

Third, the Difficulties in Emotion Regulation Scale (DERS, 36-items, [64]), validated in its French version by Dan-Glauser and Scherer [40], assesses the degree of difficulty that individuals experience in regulating emotions, and it integrates several ER domains. It is structured in six subscales and a total score. The subscales included: non-acceptance of emotional responses (six items, e.g., “*When I’m upset, I become angry with myself for feeling that way*”), difficulty in goal-directed behavior (five items, e.g., “*When I’m upset, I have difficulty getting work done*”), impulse control difficulties (six items, e.g., “*I experience my emotions as overwhelming and out of control*”), lack of emotional awareness (six items, e.g., reversed item “*I pay attention to how I feel*”, which differs from the TAS subscale “Difficulty in identifying feelings” as the lack of emotional awareness subscale focusses in the assessment of the attention’s degree that people give to emotions), limited access to ER strategies (eight items, e.g., “*When I’m upset, I believe that there is nothing I can do to make myself feel better*”) and the lack of emotional clarity (five items, e.g., “*I have no idea how I am feeling*”, which differs from the TAS subscale “Difficulty in describing feelings” as the lack of clarity subscale focusses in the different state differentiation: between emotions and between presence or absence of emotion), measured on 5-point Likert-scales with some reversed items. The Cronbach’s alpha with our sample ranged from 0.78 to 0.87.

Fourth, the participants filled out the Patient Health Questionnaire (PHQ-4, four items), measuring the levels of anxiety and depression [105]. The French version was retrieved from the Pfizer Patient Health Questionnaire (PHQ) screeners [125]. This survey is divided into two subscales, anxiety (two items) and depression (two items), and a total score, which are calculated on a scale from 0 to 3. The two items for anxiety were retrieved from the survey General Anxiety Disorder 7 [147] in its French version [115], while the two items for depression were retrieved from PHQ-9 [92], validated in French [24]. Cronbach’s alpha was 0.84 for the anxiety subscale, 0.62 for the depression subscale, and 0.81 for the total score.

#### Emotion reactivity measures

Given that emotion processes involve various emotional responses [48, 91, 108, 109], our measurements encompassed experience, expressivity, and physiological arousal.

##### Emotion experience

Throughout the laboratory study, which involved a picture viewing paradigm, participants continuously assessed their positive and negative emotional experience. To do this, they used an evaluation dial (BIOPAC Systems, Goleta, CA, USA), which allowed them to move a cursor ranging from 0 (indicating a very negative emotional experience) to 9 (indicating a very positive emotional experience, [37, 78, 151]).

##### Emotion expressivity

To examine expressivity, we used bipolar surface electromyography (EMG) on three specific facial muscle regions: the left *Corrugator Supercilii*, the left *Orbicularii Oculi,* and the left *Zygomaticus* *Major *sites. These three muscles are capable of portraying expressivity related to both negative and positive emotions. Specifically, *Corrugator Supercilii* is linked to negative emotions [97], while *Orbicularii Oculi* and the *Zygomaticus* *Major* muscles are both related to positive emotions. The electrodes were 4 mm Ag–AgCl sensors, positioned in accordance with the guidelines given by Fridlund and Cacioppo [55]. Before placing the electrodes, the skin was cleaned with alcohol and prepared with Nuprep® gel (Weaver and Cie). The electrodes were filled with Signagel (Parker Laboratories Inc.) and placed in the appropriate locations [38].

##### Physiological arousal

In the present study, Electrocardiography (ECG), SCL, as well as RR and respiratory amplitude (RA) were employed as measures of physiological arousal, assessing both the sympathetic and parasympathetic nervous system activity during the emotion elicitation. Indeed, SCL is an indicator of sympathetic nervous system activity [41], while ECG and respiration are linked to both nervous systems [71, 101]. For ECG, three sensors were used in a modified Einthoven configuration. One sensor was placed approximately 5 cm below the lower rib on the left side of the abdomen, another below the right clavicle along the midline, and a third (acting as a ground) at the level of the C7 cervical vertebrae. SCL was recorded by using pre-gelled disposable Ag/AgCl sensors placed on the thenar and hypothenar eminences of the non-dominant hand palm. Respiration was measured by using non-invasive recording belts, one placed around the waist (abdominal) and the other over the chest (thoracic, [133]). All parameters were recorded and amplified using MP150-compatible modules from Biopac Systems (Goleta, CA, USA). The acquired channels were sampled at a rate of 1000 Hz.

Finger pulse (measured with a photoplethysmography transducer on the index finger of the left hand) and skin temperature (measured with a probe on the left little finger) were recorded for another project. These two latter measurements were not further analyzed in the present study.

#### Control variables and other measures

In order to gain a more complete understanding of the participants’ characteristics, additional parameters were considered but not further analyzed in this study. Prior to their visit to the laboratory, participants were asked to complete the Edinburgh Handedness Inventory [121] in order to ascertain their degree of right-handedness. In the present study, the average score on the scale (ranging from -100 to + 100) was 96.02, with a standard deviation of 9.91. After the laboratory session, participants were additionally asked to complete several other questionnaires, the NEO Five-Factor-Inventory (NEO-FFI, 60 items, [112]) and the Berkeley Expressivity Questionnaire (BEQ, 16 items) [65], but these were not further analyzed in the present study.

### Stimuli

The use of images to induce positive and negative emotions has demonstrated to be both effective and reliable [32, 51]. Hence, an image-viewing paradigm was employed to examine affective reactivity and regulation for our different personality profile groups. A total of 144 images were presented, 72 negative and 72 positive. The images were classified into eight groups, each category containing 18 images. The four positive categories were images of babies, panoramas, sports, and cute animals, while the four negative categories were images of snakes, spiders, suffering animals, and suffering humans. 131 images were extracted from the Geneva Affective Picture Database (GAPED, [39]), while 13 additional images were added, after prior validation via an online pilot study.

### Conditions

To test the emotional reactivity towards images (H3), a condition was set-up during which participants were required to watch the image without implementing any instructed regulation (we called this condition the “*unregulated condition*”). For this, we gave participants the following instructions: “*Observe the image and let your emotion come and go naturally. Let yourself feel.*” [69]. This condition was also used for calculating the difference index, thus enabling the test of H4. As will be further described in Sect. “Data analysis”, this value was defined as a reference point to assess the efficiency of suppression and acceptance in relation to personality.

To test ER efficiency, all participants were asked to apply two ER strategies: acceptance and suppression. For the acceptance condition, the instructions were: “*Try your best to accept your emotions and try to fully live them. Do not try to avoid them and do not try to control or modify them. Avoid distracting yourself to decrease or increase your feelings. Let yourself to feel your emotions and observe which modifications the emotions trigger within you*” [38]. The primary distinction between the unregulated condition and acceptance is that the former consists of reacting as in everyday life, while acceptance implies a reflection and an observation of the felt emotions, involving an effort to welcome the affective responses.

For the suppression condition, the instructions were as follows: “*Observe the image and report what you feel but do not let the emotion affect your face and physiological reactions.*” [68].

### Procedure

**Fig. 1:**
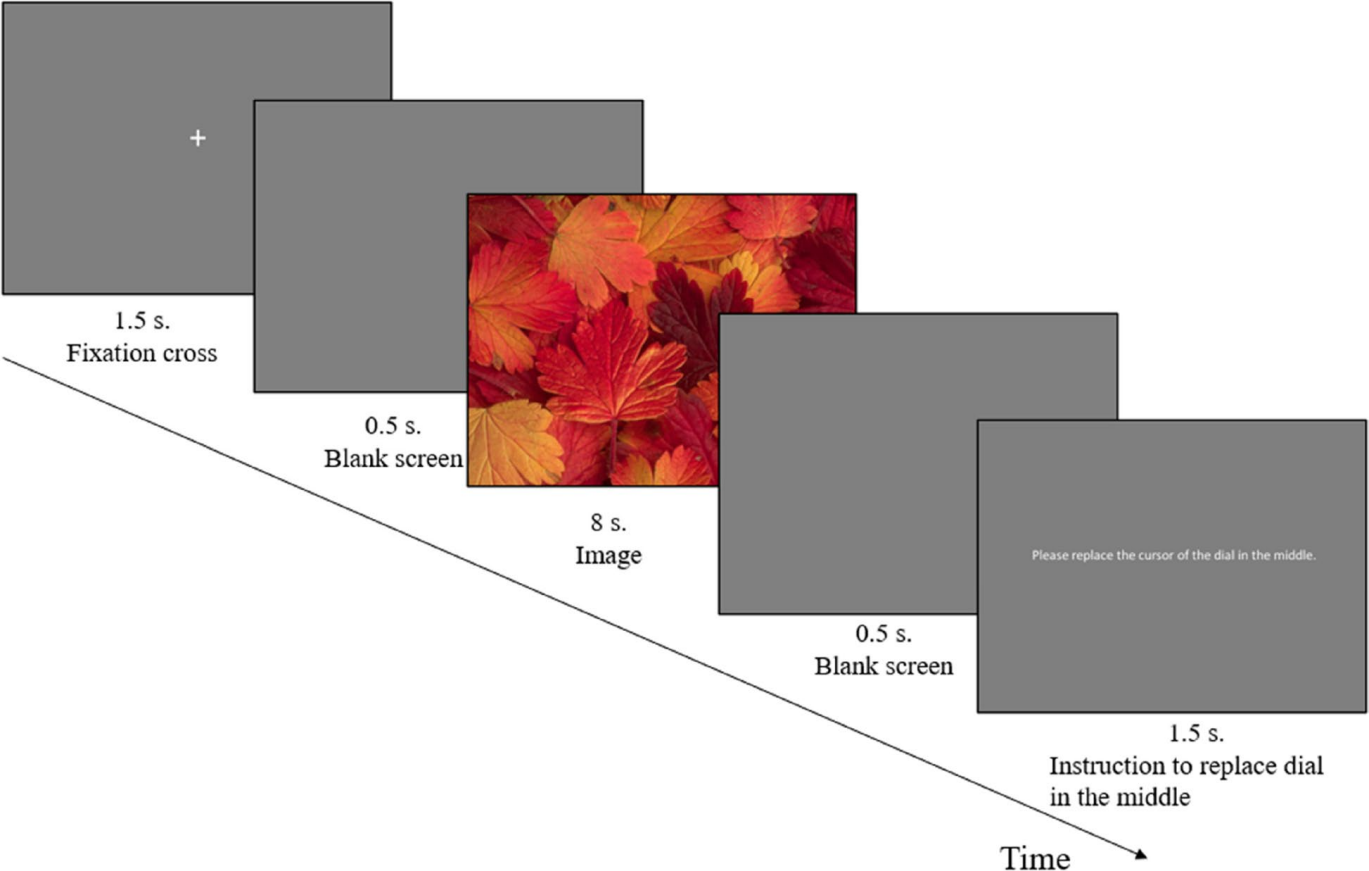
Example of a test sequence *Note*: Representation of a typical trial sequence during the experiment. Strategies instructions were displayed at the beginning of each images’ block

The protocol consisted of three stages. The first step entailed the completion of the Edinburgh Handedness Inventory [121] to confirm that they were right-handed. Subsequently, the participants proceeded to the laboratory for the second stage of the study. In the laboratory, they received detailed information about the study, including potential benefits and risks, and exclusion criteria were repeated. If they were eligible and agreed to participate they signed an informed consent. The main experiment began after the placement of the physiological sensors. All instructions were presented on a computer screen. During the experiment, each participant was asked to accept, suppress, or just watch the images, which were presented in a semi-randomized sequence. The images were shown in nine blocks, three per condition, with 16 images in each block. At the start of each block, participants were given instructions on the strategy they were required to apply. For each trial, participants saw, in sequence, a fixation cross, a blank screen, the image itself (displayed for 8 s), and another screen asking to reset the dial for the next presentation. During each trial, participants were asked to continuously rate their emotional experience using the rating dial in front of them (see trial sequence details in Fig. 1). At the end of this task, which lasted approximately 45–50 min, the physiological sensors were removed, and participants were debriefed. The third and final step was conducted within seven days of the laboratory visit. Participants were asked to complete the questionnaires, by using a specific URL received during the laboratory session. During this phase, they completed the PID-5-SF, the TAS, the PHQ-4, the DERS, and the PANAS. They also filled the NEO-FFI and BEQ at this stage. At the end of the questionnaire phase, participants were fully debriefed. The procedure was reviewed and authorized by UNIL-SSP ethics committee (approval number: C_SSP_112020_00006), in accordance with the current national legal requirements (Ordinance on Human Research) and the latest version of the declaration of Helsinki.

### Data pre-processing

Personality trait scores from the PID-5-SF were standardized (z-scores) before conducting the cluster analysis following Rossi et al. [139] and Bastiaens et al. [17]. From the raw scores, we first reversed the scores when appropriate and, then, total scores and subscales were calculated by averaging the item values to make the subscale scores comparable. All data related to experience, expressivity, and physiological arousal were processed using Acqknowledge 4.4 software (Biopac Systems, Goleta, CA). Recorded channels were band-pass filtered in specific frequency ranges to improve the signal-to-noise ratio (20–500 Hz for EMG, 0.5–35 Hz for ECG and 0.05–1 Hz for respiration). Manual scanning and correction of data artifacts were performed to ensure data quality [48, 151, 152]. To minimize tonic response effects, changes in the physiological and expressive channels were compared to a baseline period of 3.5 s, taken before each picture viewing [152]. This baseline period was used to obtain the relative change in parameters following each image. Parameter changes were averaged over the entire 8 s duration of each image display, as some prior studies similarly performed (e.g., [69, 116]). Trials were classified according to the regulated conditions (suppression and acceptance), and each condition was compared to the unregulated condition to examine the efficiency of the strategies (see Data Analysis section). More information about the specific emotional response signal reduction are described below.

#### Emotional experience

To determine the participants’ emotional response to each trial, we exported the ratings and calculated the average value for each trial. We took the cursor position before each trial as the starting point for determining the intensity of the emotional experience felt during that trial. We classified any value below this position as a negative feeling and any value above as a positive feeling. To make the ratings more understandable, we converted them into a measure representing the distance travelled between the starting point and the cursor position reached during each trial. We then rescaled the score to cover a range 0 to 100. For each valence, the final values for experience hence ranged from 0, representing no emotional experience, to 100, representing an extreme intensity of emotional experience felt during the trial.

#### Emotion expressivity

To analyze EMG signals, we rectified and smoothed them at 5 Hz. Since each individual has a different contractile capacity, we transformed each EMG value into a percentage of contraction relative to the corresponding trial’s antecedent level. We did this by dividing the recorded voltage by the voltage recorded during the 3.5 s preceding the trial, then multiplying it by 100. This method allowed for more accurate comparison and evaluation, as suggested by De Wied et al. [43] and Van Boxtel [159]. Negative expressivity was measured with the *Corrugator* site values. Positive expressivity was measured with the average of the *Orbicularii* and *Zygomaticus* site values, since they were positively correlated (r = .610, *p* < .001).

#### Physiological arousal

HR was determined by calculating the interbeat interval (the interval between consecutive R waves) based on the ECG channel. One participant was excluded from the HR analysis because the signal was too noisy. SCL was exported as the average value for each trial. Nineteen participants were excluded from the SCL analysis because they were non-responders, making their physiological reactivity irrelevant for trial-by-trial examination [57, 89]. RR was obtained in cycles per minute (c/min). RA was determined by calculating the voltage difference between the point of maximum inspiration and the point of maximum expiration. For both respiratory parameters (rate and amplitude), thoracic and abdominal values were correlated, RR_Negative_ r = .374, *p* < .001, RR_Positive_ r = .203, *p* = .039, RA_Negative_ r = .203, *p* = .040, RA_Positive_ r = .338, *p* < .001. Consequently, signal values for thoracic and abdominal channels were averaged together for each participant and each valence. All physiological response channel data were measured as the change from the baseline level in each trial.

### Data analysis

IBM SPSS Statistics software (version 28) was used for all analyses. As a first step, we performed analyses to determine personality profiles (H1). We used the k-means clustering to define personality groups. We preferred this approach because, unlike other analyses like the Latent Profile Analysis, it allowed a good balance of the participants between clusters. Furthermore, it allowed to identify the clusters on predefined criteria, permitting us to target profiles with opposite traits [139].

To better define the main features of the profiles in terms of emotional symptoms and difficulties, we analyzed how our personality profiles differed in their mean total scores an subscales of the TAS, DERS and PHQ (H2). For the PANAS only the Negative Affect and Positive Affect subscales were analyzed (no total score). To do this, we ran independent t-test analyses. P-values were corrected with Holm-Hochberg procedure.

To assess emotional reactivity between maladaptive profiles (H3), we compared the distribution of affective responses during unregulated trials with a 100-centered (corresponding to expressivity at baseline level) or a 0-centered (all other parameters at baseline level) distributions with one-sample t-tests in each profile and we then highlighted the differences between profiles. For experience and expressivity, we chose to consider the whole trial duration because these reactions are immediate and stable over time. Since physiological parameters can have a shorter reactivity, we decided for these parameters to focus only on the time frames that showed a physiological reactivity within a 4 s range. To test our fourth hypothesis, and for each of the parameters studied, we calculated a difference index (DI), defined as the difference between the unregulated condition and each of the strategies, in the direction directly highlighting regulation efficiency. DI is hence interpreted with the following rationale: all reactions for which the DIs are significantly below 0 are considered as not reflecting ER efficiency, and even witnessing a deleterious reaction as they represent an increase in emotional arousal when regulation is applied. On the other hand, the reactions that have values significantly above 0 indicate that the regulation is efficient. Zero (or close to 0) values indicate no changes in emotional responses during the regulated condition, as compared to the unregulated trials. Such values are also assimilated to strategy inefficiency.

The test of H4 was performed with repeated-measures ANOVAs with a within-subject factor, *Condition* (two levels: Acceptance DI, Suppression DI) and a between-subject factor, *Personality* (number of cluster groups). When the interaction was significant, post-hoc analyses were performed. Independent t-tests were performed to compare the efficiency of each strategy between profiles, and paired t-tests were performed between acceptance and suppression within each personality profile. Note that for testing ER efficiency, repeated-measures ANOVAs were conducted only on parameters where the emotional induction was successful in both profiles since there is no point in considering regulation efficiency in the absence of emotional reaction. When a parameter resulted in a significant emotional induction in only one profile, a paired t-test was performed to test whether one strategy was more efficient than the other in that particular profile.

## Results

### Personality profiles

#### Determination of personality profiles (H1)

**Fig. 2:**
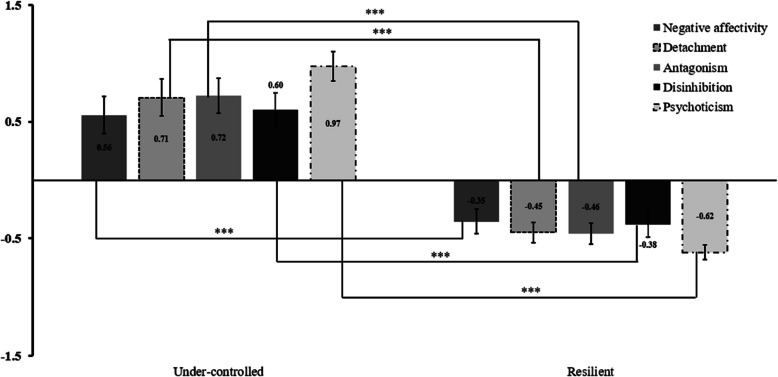
Personality clusters based on PID-5 z-scores *Note*: Standardized trait scores for the two resulting clusters, “Resilient” and “Under-controlled”. The asterisks linking the bars represent the paired t-tests of the same trait between profiles. Dispersion is represented by the Standard Error of the Mean, ***p < .001

We identified two personality profiles (Fig. 2). Following Bastiaens et al. [17], we labeled the first profile as “Under-controlled” (N = 40) and the second one as “Resilient” (N = 63). For the first cluster, the within-cluster sum of squares was 163.78, while for the second one it was 173.47. In Under-controlled profile, all traits were higher than in the Resilient profile, t_(101)_ = 5.01 to 11.32, *p* < .001, d = 1.01 to 2.26. These results were consistent with those of Trentini and Dan-Glauser [156].

### Characterizing emotional symptoms and difficulties of personality profiles (H2)

**Fig. 3:**
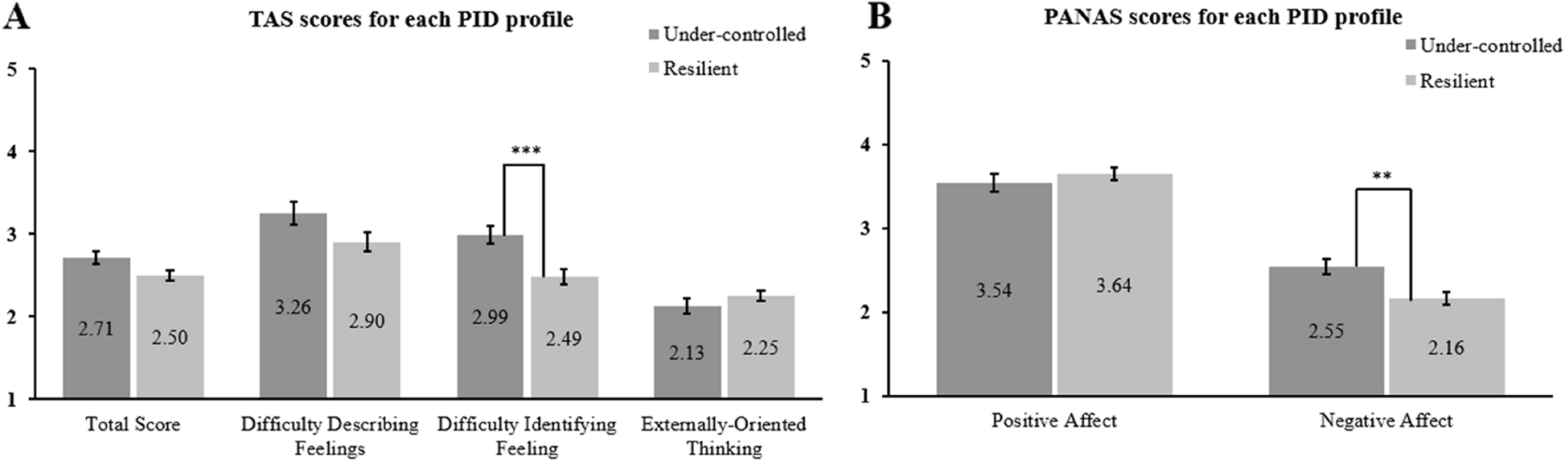
Comparison of the personality profiles on the TAS and PANAS scores *Note*: Panel **A**: average for the TAS total score and each of its subscales for each profile, scale ranges from 1 to 5. Panel **B**: average results for the PANAS for each profile and each subscale, scale ranges from 1 to 5. Dispersion is represented by the Standard Error of the Mean. Links between bars indicate significant differences between profiles, **p < .01, *p < .05

On the TAS, Under-controlled individuals scored on average higher in “Difficulty in identifying feelings”, t_(101)_ = 3.59, corrected *p* < .001, d = 0.73. However there were no significant difference in the total score, t_(101)_ = 2.23, corrected *p* = .056 d = 0.45, and in the subscales of “Difficulties of describing feelings”, t_(101)_ = 1.98, corrected *p* = .147, d = 0.40 and “Externally-oriented thinking”, t_(101)_ = -1.11, *p* = .268, d = -0.22 (Fig. 3, Panel A).

On the PANAS, Under-controlled profile had higher levels of negative affect than the Resilient one, t_(101)_ = 3.11, corrected *p* = .002, d = 0.63, but no significant difference was found for levels of positive affect, t_(101)_ = -0.83, *p* = .406, d = -0.17 (Fig. 3, Panel B).

**Fig. 4:**
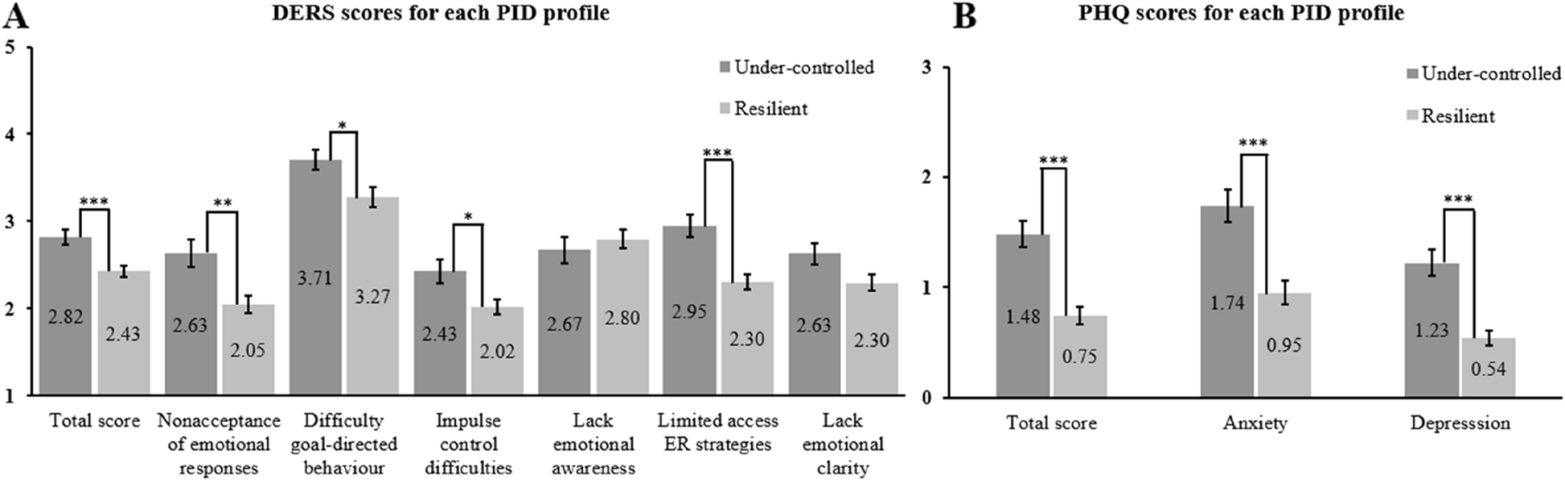
Comparison of the personality profiles on the DERS and PHQ scores *Note*: Panel **A**: results for the DERS total score and each of its subscales for both personality profiles, scale ranges from 1 to 5. Panel **B**: PHQ scores for each profile (total score and each subscale), scale ranges from 0 to 3. Dispersion is represented by the Standard Error of the Mean. Links between bars indicate significant differences between profiles, ***p < .001, *p < .05

For the DERS, significant results between profiles were found on the total score, t_(101)_ = 3.73, corrected p < .001, d = 0.75, on the “Nonacceptance of emotional responses” subscale, t_(101)_ = 3.26, corrected *p* = .006, d = 0.66, on the “Difficulty goal-directed behavior” subscale, t_(101)_ = 2.55, corrected *p* = .036, d = 0.52, on the “Impulse control difficulties” subscale, t_(101)_ = 2.61, corrected* p* = .05, d = 0.53, and on the “Limited access to ER strategies” subscale, t_(101)_ = 4.23, corrected *p* < .001, d = 0.85. No significant difference was found on the “Lack of emotional awareness” subscale, t_(101)_ = -0.74, *p* = .458 d = -0.15, nor on the “Lack of emotional clarity” subscale, t_(101)_ = 2.22, corrected *p* = .168, d = 0.45 (Fig. 4, Panel A).

Finally, for the PHQ, we found higher scores in Under-controlled people on the total score, t_(101)_ = 5.19, corrected *p* < .001, d = 1.11, the anxiety score, t_(101)_ = 4.48, corrected *p* < .001, d = 0.91 and the depression score, t_(101)_ = 4.81, corrected *p* < .001, d = 1.05 (Fig. 4, Panel B).

### Emotional reactivity for the different personality profiles (H3)

We then examined whether the profiles differentially reacted to the emotional induction procedure (unregulated condition only). Table 1 represents the experience, expressivity and physiological parameter’s change in the unregulated condition, for both negative and positive images and for both personality clusters.

**Table 1:** Reactivity measures for each profile, emotional parameter and valence level

Emotional response	PID-5 M, Cohen’s *d*		Difference between clusters
	**Under-controlled**	**Resilient**	**t, Cohen’s *d***
**Experience**			
Negative	34.90, 2^***^	42.10, 2.6^***^	-2.13, -.43^*^
Positive	33.05, 2.2^***^	37.60, 2.5^***^	1.45, .30
**Expressivity** (%)			
Negative	176.90, .46^**^	154.52, .78^***^	.93, .19
Positive	172.86, 1.14^***^	168.31, .85^***^	.30, .06
**Physiological Arousal**			
HR (Δbpm)			
Negative	.38, .19	-1.02, .66^***^	1.82, .37
Positive	.13, .12	.02, .02	.48, .10
SCL (ΔμS)			
Negative	.03, .35	.02, .15	.42. -.37
Positive	-.06, -.60^*^	-.03, -.28	-.11, -.26
RR (Δcpm)			
Negative	.11, .20	.13, .24	-.16, -.03
Positive	.24, .45^**^	.18, .42^**^	.63, .13
RA (ΔμV)			
Negative	.05, .31	-.01, -.03	1.64, .33
Positive	-.1, -.30	-.01, -.11	-1.96, -.40

*Note*: Bold text represents significant inductions (*p* < .05). bpm = beats per minutes, μS = micro-Siemens, cpm = cycles per minute, μV = micro-Volt, ***p < .001, ** *p* < .01, *p < .05.

### Efficiency of acceptance and suppression as a function of personality profiles (H4)

The table below (Table 2) shows the efficiency of both strategies, as a function of personality profiles.

**Table 2:** Strategy efficiency (measured with the DI for each parameter and each valence level) as a function of personality profiles

Emotional response	PID-5 M, Cohen’s *d*			
	**Under-controlled**		**Resilient**	
	Acceptance	Suppression	Acceptance	Suppression
**Experience**				
Negative	1.38, .22	.27, .04	.32, .06	-3.01^***^, -.56
Positive	-1.03, -.16	.21, .03	-.62, -.10	1.45^*^, .25
**Expressivity** (%)				
Negative	-18^**^, -.48	60^*^, .37	-10.4^*^, -.27	39.61^***^, .65
Positive	-19.31^*^, -.37	60.98^***^, .1.13	-18.18^**^, -.41	59.38^***^, .83
**Physiological Arousal**				
Heart Rate (Δbpm)				
Negative			.42^*^, -.25	-.09, .06
Positive				
Skin Conductance (ΔμS)				
Negative				
Positive	.02, .17	-.01, -.13		
Respiratory Rate (Δcpm)				
Negative				
Positive	.18, .30	-.07, -.11	-.03, -.05	-.04, -.08
Respiratory Amplitude (ΔμV)				
Negative				
Positive				

*Note*: Efficiency of each strategy for the different personality profiles, ***p < .001, **p < .01, *p < .05. Efficiency is indicated in bold. These results were obtained from one-sample t-test analyses on DI values, only for parameters for which induction was successful (see Table 1)

#### Experience

**Fig. 5:**
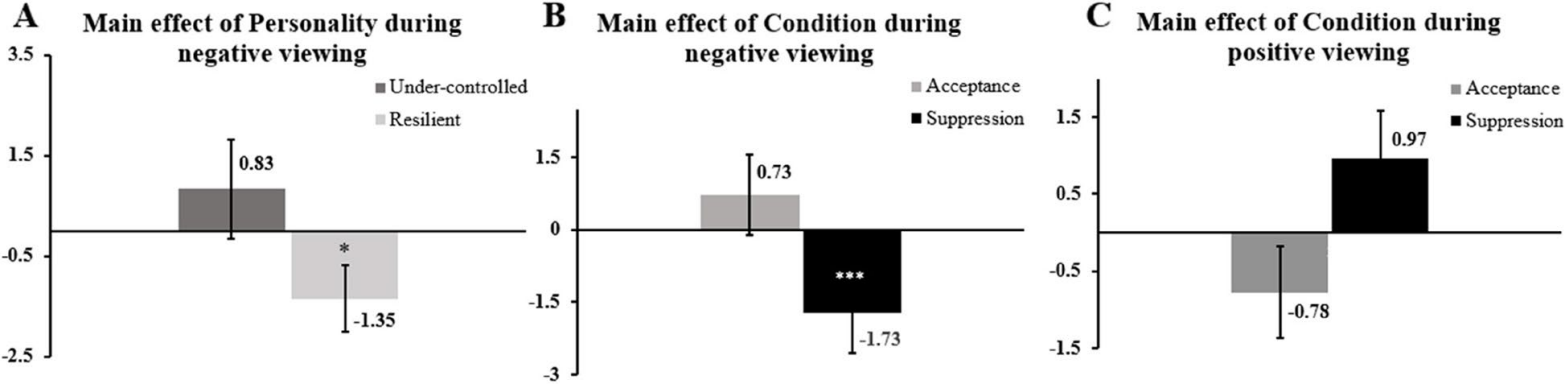
DI values for emotional experience during negative (**A** and **B**) and positive (**C**) viewing *Note*: Dispersions are shown with the Standard Error of the Mean. Asterisks inside bars represent efficiencies for each profile (**A**) or strategy (**B** and **C**), ***p < .001, *p < .05

With respect to experience during negative viewing, the main effects of Personality, *F*_(1;101)_ = 5.105, *p* = .026, η_p_^2^ = .05 (Fig. 5, Panel A) and Condition, *F*_(1;101)_ = 11.913, *p* < .001, η_p_^2^ = .11 (Fig. 5, Panel B) were significant; while the interaction between Condition and Personality was not significant, *F*_(1;101)_ = 2.971, *p* = .088, η_p_^2^ = .03. Subsequent post-hoc analyses related to the main effect of Personality revealed a significant difference between profiles during negative viewing, t_(101)_ = 2.26, *p* = .026, d = 0.46, meaning that Resilient group significantly experienced more negative emotions, independently from the regulatory strategy applied, t_(62)_ = 2.40, *p* = .019, d = 0.30. This is not the case for the Under-controlled group, which reported no significant difference in experience t_(39)_ = -1, *p* = .323, d = 0.16. Concerning post-hoc analyses for the main effect of Condition, there was a significant difference between acceptance and suppression, t_(102)_ = 3.89, *p* < .001, d = 0.38. Suppression resulted significantly counterproductive by triggering more negative experience, t_(102)_ = -2.98, *p* = .004, d = 0.13, while acceptance did not impact experience, t_(102)_ = 1.29, *p* = .197, d = 0.30.

Regarding the same parameter during positive viewing, only the main effect of Condition was significant, *F*_(1;101)_ = 8.392, *p* = .005, η_p_^2^ = .08 (Fig. 5, Panel C). The interaction Condition x Personality, *F*_(1;101)_ = 0.509, *p* = .477, η_p_^2^ = .005 and the main effect of Personality, *F*_(1;101)_ = 0.566, *p* = .454, η_p_^2^ = .01, were not significant. Post-hoc analyses for the main effect of Condition revealed a significant difference between acceptance and suppression, t_(102)_ = 3.14, *p* = .002, d = 0.31, showing that suppression (Mean = 0.97) was more efficient than acceptance (Mean = -0.78). However, neither acceptance, t_(102)_ = -1.31, *p* = .193, d = 0.13, nor suppression, t_(102)_ = 1.60, *p* = .113, d = 0.16 were significantly impacting positive experience during the viewing.

#### Expressivity

**Fig. 6:**
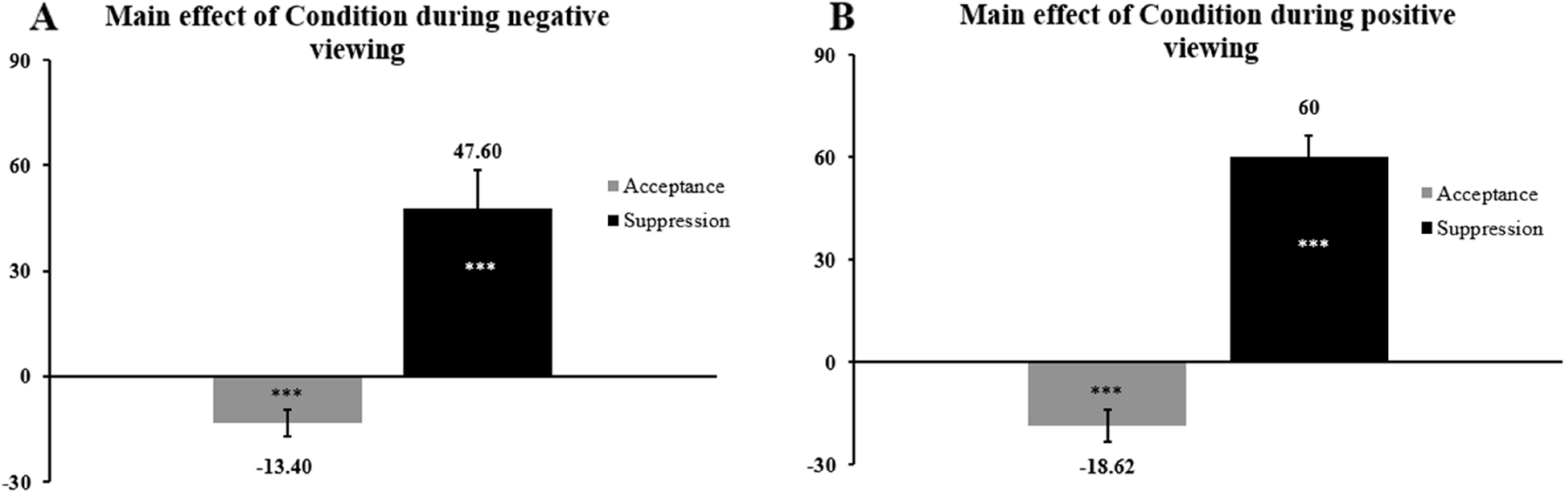
DI values of expressivity during negative (Panel **A**) and positive (Panel **B**) viewing *Note*: Dispersions are shown with Standard Error of the Mean. Asterisks inside bars represent post-hoc analyses measured with one-sample t-tests (efficiency testing) , *** = *p* < .001

Regarding expressivity, the *Corrugator* muscle region during negative viewing showed a significant main effect of Condition, *F*_(1;100)_ = 28.355, *p* < .001, η_p_^2^ = .22 (Fig. 6, Panel A), but no significant interaction Condition x Personality, *F*_(1;100)_ = 1.353, *p* = .248, η_p_^2^ = .01, nor a main effect of Personality, *F*_(1;100)_ = 0.303, *p* = .584, η_p_^2^ = .003. In the post-hoc analysis of the main effect of Condition, both acceptance, t_(101)_ = -30.40, *p* < .001, d = 3, and suppression, t_(101)_ = -4.81, *p* < .001, d = 0.47, showed a significant level of parameter change, indicating inefficiency of acceptance and strong efficiency of suppression.

Similar results were found for expressivity during positive viewing (Fig. 6, Panel B). Indeed, the main effect of Condition resulted significant, *F*_(1;101)_ = 98.484, *p* < .001, η_p_^2^ = .48, but there were no significant results in the interaction Condition x Personality, *F*_(1;101)_ = 0.028, *p* = .867, η_p_^2^ = .00 and in the main effect of Personality, *F*_(1;101)_ = 0.001, *p* = .977, η_p_^2^ = .00. In the subsequent analysis regarding the main effect of Condition, we tested the efficiency of each strategy with one-sample t-tests. Acceptance was t_(102)_ = -3.96, *p* < .001, d = -0.39, and suppression was, t_(102)_ = 9.4, *p* < .001, d = 0.93, meaning that acceptance reinforced the positive expressivity whereas suppression successfully reduced it.

#### Physiological arousal

**Fig. 7:**
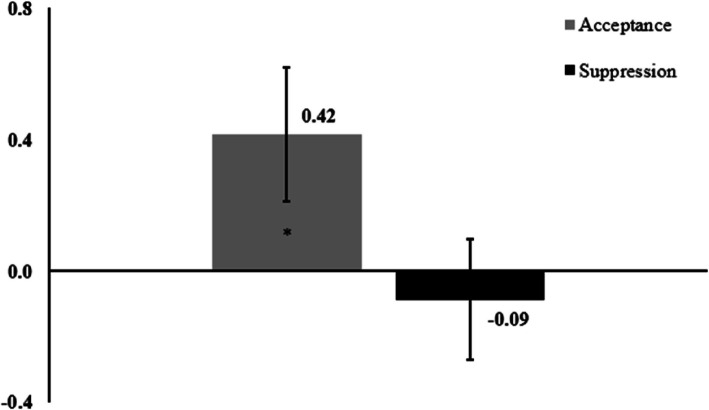
DI values of acceptance and suppression HR change in Resilient people during negative viewing *Note*: Dispersions are shown with Standard Error of the Mean. Asterisks inside bars represent post-hoc analyses measured with one-sample t-tests (strategy efficiency), * = *p* < .05

The results of a paired t-test performed on the HR measure of the Resilient profile during negative viewing showed significant results, t_(62)_ = 2.13, *p* = .037, d = 0.27 (Fig. 7). As also shown in Table 2, the one sample t-tests revealed an efficiency of acceptance, t_(62)_ = 2.04, *p* = .045, d = 0.26, but not of suppression, t_(62)_ = -0.48, *p* = .636, d = -0.06, which does not seem to alter HR. Comparison of strategy efficiency for SCL in Under-controlled people during positive viewing showed non-significant results, t_(27)_ = 1.42, *p* = 0.166, d = 0.27. For RR during positive viewing, the main effect of Condition, F_(1;101)_ = 3.931, *p* = 0.05012, η_p_^2^ = 0.04, the Condition x Personality interaction, *F*_(1;101)_ = 3.303, *p* = 0.072, η_p_^2^ = 0.03, and the main effect of Personality, *F*_(1;101)_ = 0.789, *p* = 0.376, η_p_^2^ = 0.008, were not significant.

## Discussion

In this study, we examined three main aspects of emotion and ER processes in relation to maladaptive personality: 1) differences between personality profiles in how individuals generally deal with emotions, as well as the presence of potential symptoms such as anxiety, depression and alexithymia; 2) the extent to which profiles react in terms of emotional experience, expressivity and physiological arousal when confronted with emotional stimuli, and 3) the differential efficiency of acceptance and suppression strategies for different personalities. Considering the novelty of some of the topics addressed in this study, several hypotheses were exploratory and are further discussed below.

### Characterization of personality profiles: potential risk factors and emotion difficulties

Regarding the first hypothesis on the nature of personality profiles, we found two groups, the Resilient and the Under-controlled, confirming our assumptions. The Resilient profile is characterized by low scores in all maladaptive traits and, on the other hand, the Under-controlled presented high scores in all maladaptive traits. These results are consistent with our previous study [156] and similar to Bastiaens et al. [17]. Furthermore, the dichotomy between our profiles may be an advantage as they well describe the more adaptive side of MPTM (Resilient) and the other side, characterized by higher pathological values (Under-controlled), as evidenced by Rossi et al. [139].

Then, we characterized the personality profiles in terms of emotional symptoms and difficulties (H2). The in-depth analysis of personality groups aimed at better targeting the main emotional problems of these profiles and informing future hypotheses regarding intervention tailoring in clinical populations. Regarding alexithymia, the Under-controlled individuals reported greater difficulties in identifying feelings than the Resilient ones, partially confirming our hypothesis. It could be then possible that, when Under-controlled people perceive an emotion, they may be less able to understand what they are experiencing compared to Resilient people, potentially leading them to react inappropriately to the emotional stimulus [42]. This is consistent with Garofalo et al. [56], who found significant correlations between this subscale and all maladaptive traits in community-dwelling women, and showing that the difficulty of identifying feelings strongly predicts negative outcomes [132]. Based on these results, it could be hypothesized that these inappropriate emotional reactions may contribute to the enhancement of ER difficulties, as Koppelberg et al. [88] also suggested.

Interestingly, however, there was no significant difference between profiles on any of the other subscales after correcting for multiple testing. There may be statistical factors that could explain this absence, such as sample size. Furthermore, even if the TAS is widely applied in literature, it would be interesting to test alexithymia in the future with an alternative instrument, for example, the Perth Alexithymia Questionnaire [128]. This instrument provides a similar assessment of alexithymia construct as the TAS [129], but its main advantage lies in the discrimination between negative and positive emotions [128]. In future studies aiming to replicate our results, this survey would allow to explain the possible discrepancies within parameters and between valences. Additionally, the valence discrimination would give insights into the presence and the extent of potential differences and deficits in identifying and describing emotions in maladaptive personality profiles as a function of emotion valence. For instance, it could be hypothesized that maladaptive personality groups have more difficulties in identifying and describing negative emotions than positive ones, which could explain why these groups may have fewer benefits from the application of ER strategies. A final reason to explain this result is related to the nature of the studied population. We tested healthy, undergraduate participants, and it can be possible that alexithymia levels are lower in this population, as Loftis et al. [104] also reported in a similar sample. When clinical populations were considered, several studies revealed that individuals with psychopathy [22] and BPD [44] presented alexithymia and, in Andersen et al. [11], the clinical group scored higher than the non-clinical one. Thus, in the future, it would be interesting to test a clinical group to assess alexithymia according to personality profile.

Regarding affect, the Under-controlled group experienced higher negative affects on average than the Resilient one, confirming prior results [94, 127, 135]. Nevertheless, it is interesting to note the score similarity in both profiles regarding positive affects. Since positive affects can be considered as an indicator of resilience and a shield from stressors [158], we had expected that Under-controlled individuals would experience less positive affects. Since this is a non-clinical population, it is possible that positive affects are experienced regularly by both profiles and that personality group does not influence positive emotions in our sample. It would be interesting to test people with higher scores on maladaptive traits to see if lower positive affects are noticed for more extreme populations. In the present study, we considered that negative and positive affect operate as partially independent dimensions (Watson & Tellegen, 1985), rather than opposite poles of a single continuum. This helps explain why we were able to show that the Under-controlled profile shows elevated negative affects while maintaining average levels of positive affects. This pattern mirrors what found in the literature on personality focused on the FFM, where neuroticism and extraversion — which are also associated with negative and positive affects, respectively — are treated as distinct traits [33, 111]. Furthermore, this comparison with personality highlights the complexity of the phenomena, indicating that a simplistic interpretation of affects (e.g., if positive affect is high then negative is low) or of personality (e.g., if a person is extraverted then it cannot show high levels of neuroticism), does not reflect all aspects of an individual. Therefore, similarly as neuroticism and extraversion can coexist simultaneously, the presence of high negative affects in this group does not preclude the experience of positive affects, especially in non-clinical populations. In terms of ER difficulties, the Under-controlled cluster showed higher values than the Resilient group in the total score, as well as in the “Non-acceptance of emotional responses”, “Impulse control difficulties”, “Difficulty goal-directed behavior” and “Limited access to ER strategies” subscales. Overall, these findings are consistent with previous results in a non-clinical population [9, 127]. In those cases, analyses were conducted on the correlations between traits and DERS subscales, and ER difficulties were positively correlated with all maladaptive traits. In particular, the “Non-acceptance of emotional responses” subscale can be considered a specific indicator of all pathological personality clusters in the inability to manage emotions, and a potential predictor of higher vulnerability to suffer from depression and anxiety [1]. In addition, higher scores in “Impulse control difficulties”, “Difficulty goal-directed behavior”, and “Limited access to ER strategies” indicate a general negative behavior in the Under-controlled cluster when ER is needed. By avoiding negative stimuli [134] or refusing to feel negative emotions, Under-controlled people may have difficulties controlling their impulsive tendencies [160]. This leads the Under-controlled group to perceive that they cannot change their emotional state and that they cannot provide any strategy to regulate emotions (“Limited access to ER strategies”). Altogether, this cognitive situation negatively obscures the final emotional response, leading to difficulties in directing their behavior towards the negative stimulus. Another point to consider is the absence of significant differences between profiles for “Lack of emotional clarity” and “Lack of emotional awareness”. This result is similar to that of Igra et al. [79], who found no significant differences between the clinical and the healthy group in “Lack of emotional awareness”. Besides the possible low power due to the sample size, it is possible that, from a conceptual perspective, “Lack of emotional clarity” and “Lack of emotional awareness” subscales may be more appropriate for detecting aspects related to emotion identification rather than related to ER [114] and, hence, less adequate for understanding the degree of ER difficulties. Second, there is a body of research that indicated poor psychometric properties of the “Lack of emotional awareness” subscale (e.g., [15]), mainly due to its structure, all items being reversed-scored. This casts a doubt about the usefulness of this subscale in assessing difficulties in ER, suggesting that a removal of this subscale would be more appropriate [123]. Finally, although the “Lack of emotional awareness” subscale seems at first glance to be related to “Difficulties in identifying feelings” and “Difficulties in describing feelings” TAS subscales, some studies have reported contrasting categorization, leading to confusion over its definition. Indeed, Lawlor et al. [99] grouped these TAS subscales under the “Clarity” dimension, while Kimhy et al. [84] classified the same subscales under the “Awareness” dimension. Hence, given the criticisms and the discrepancies surrounding the “Lack of emotional awareness” subscale, we suggest a cautious interpretation of this latter finding.

Regarding the tendency to suffer from anxiety and/or depression, Under-controlled group scored higher than the Resilient one, on average, on all subscales and on the total score, confirming our expectations. This is consistent with previous studies [35, 172] and these results confirm the higher propensity of maladaptive personality to suffer from internalizing disorders such as anxiety and depression.

Overall, our main hypotheses were confirmed, and our results showed that people with high maladaptive traits experience stimuli more negatively, with more problems in identifying, describing, and regulating feelings. From a clinical perspective, working on how emotions are perceived and consequently regulated could lead to improvements in many other facets of the individual. In that matter, dialectical behavior therapy (DBT) could be used. This type of treatment is used for several disorders, particularly BPD, and is close to the modal model [70]. Especially for Under-controlled people, DBT could be a powerful tool to treat impulsivity, emotion dysregulation and low tolerance to distress [106] as it places particular emphasis on ER. Moreover, biological, expressive and cognitive aspects are involved to change the “pervasive” dysfunctional perspective patients have towards emotions [118]. Furthermore, as DBT includes several modules, recent findings suggest that the ER module alone may be sufficient to yield significant improvements in regulatory functioning [28], which could make it a more accessible and targeted approach, particularly for non-clinical or subclinical populations. Therefore, starting from the development of ER skills and distress tolerance techniques, Under-controlled people would be able to respond to emotional stimuli in a more organized and reasoned way, providing broader access to ER strategies.

### Emotional reactivity of the different profiles

In the analysis of emotional reactivity between personality clusters in emotional responses, we found interesting results in some parameters, which, in some cases, ran against our predictions.

In experience, both personality groups showed a significant reactivity to both negative and positive images. Of particular note was the significantly higher reactivity of the Resilient group in experience towards negative images, as compared to the Under-controlled people. As the PANAS results show, the Resilient group felt less negative affect, which could lead them to be more touched and triggered by negative stimuli in comparison to Under-controlled people, who are more frequently exposed to negative affects, as similarly Herpertz et al. [76] found in physiological arousal. The presence of a habituation effect to negative stimuli is thus possible, which could explain this result, but more analyses are needed to verify this hypothesis.

Similar results were found for expressivity, both profiles showing significant reactivity in both valences, but no significant difference between clusters was found. This lack of difference could indicate the presence of the interplay between personality and the studied parameters already discussed in Trentini and Dan-Glauser [156], where the influence of personality seems stronger for some parameters, while it seems less detectable for others. Furthermore, as this is a non-clinical population, it may be plausible to find fewer differences in expressivity, explaining the contrast with Renneberg et al. [131], who found reduced expressivity in a clinical population.

Regarding physiological parameters, only HR for negative viewing in the Resilient profile, RR for positive viewing in both profiles, and SCL for positive viewing in the Under-controlled profile, showed significant emotional reactivity. These results confirmed what we found in Trentini and Dan-Glauser [156], where not all parameters were reactive to emotional stimulation, and with some differences between personality clusters. For instance, the Resilient profile presented a significant decrease in HR values, which was not found in the Under-controlled group. This result indicates a stronger orientation of the attention towards the negative stimulus [21] for the Resilient profile. These results could also be an indication that Resilient people, considered as the adaptive profile in the MPTM, allocate more resources to emotion processing, and are therefore able to reduce the impact of negative stimuli on HR (with acceptance, see Sect. "Personality profiles and ER efficiency" below). Concerning SCL in the Under-controlled group, this is consistent with a previous study [156], where non-significant results were found. This was also similar to those of Herpertz et al. [76] on BPD patients, whose population share common aspects with our Under-controlled group. In their study, they found a hyporeactivity of skin conductance activity and they suggested that this was due to a probable lack of attentional processing. Therefore, we can hypothesize a similar reason for our results, but further analyses on the attentional aspects are needed to verify this. RR was reactive for positive viewing in both profiles but not for the negative one. Subsequent analyses with personality as a between-factor revealed that this was independent of personality profile. Although this result might seem counterintuitive at first glance, it is actually consistent with Shiota et al. [144], who showed an increased RR during the viewing of positive images, especially the most arousing ones [90]. Finally, the absence of reactivity in RA, which also occurred in Trentini and Dan-Glauser [156], confirms the inadequacy of RA as a parameter to detect differences between personality profiles, at least regarding the profiles evidenced here. Overall, these results confirmed the hypothesis previously discussed in Trentini and Dan-Glauser [156] that, according to personality profiles, there could be a different physiological pattern in emotional reactivity, leading to the activation of some parameters but not others. This highlights underlying differences between personalities in terms of autonomic nervous system functionning. In the case of Under-controlled people, it may indicate a co-inhibition of sympathetic and parasympathetic nervous system, as Thomson [150] suggested. In other words, it describes the presence of low sympathetic and parasympathetic activity, and the low SCL values and the lack of reactivity in HR in the Under-controlled group could reflect this phenomenon. However, this hypothesis should be further tested with other physiological parameters, such as salivary or hemodynamic parameters, to better define the singular sympathetic and parasympathetic activity of the different personalities [7]. Another aspect that these results highlight is that the valence of emotions can be considered as a factor likely to influence physiological reactivity. For instance, with SCL and RR, we found a significant reactivity during positive viewing but not during the negative one, confirming the presence of a physiological activation pattern as a function of emotional valence [90].

### Personality profiles and ER efficiency

Regarding experience during negative viewing, we found significant main effects of Condition and Personality, but no interaction between these factors. First, this means that, regardless of personality, suppression is deleterious while acceptance has no impact on experience. The non-significant results of acceptance are consistent with Dan-Glauser and Gross [38], where acceptance produced no significant change in experience. Regarding suppression, it has previously been classified as a maladaptive strategy, which is consistent with our results. We can therefore assume that neither acceptance nor suppression could be efficient enough to reduce negative experience in the short term, regardless of personality. This confirms our assumptions for suppression, but not for acceptance. While acceptance is often described as adaptive in the literature [157], its benefits may not manifest in immediate reductions of negative affects. As suggested in mindfulness-based frameworks (e.g., Acceptance and Commitment Therapy, [75]), acceptance may primarily promote long-term emotional well-being by reducing experiential avoidance rather than short-term distress. This encourages future studies with longer-term follow-ups and potentially prior training. Second, regardless of the implemented strategy, negative experience varies according to personality. None of the profiles did obtain benefits in reducing negative experience, especially the Resilient group, where the strategies were even generally increasing the negative experience, as compared to the unregulated condition. For positive experience, only the main effect of Condition was significant. Despite the significant difference between the strategies, none of them seemed actually efficient. Regarding experience, we can highlight the absence of significant results for acceptance in both personality profiles.

Concerning expressivity, the hypotheses were partially confirmed. Indeed, as previously shown in Trentini and Dan-Glauser [156], personality has little overall influence on the expressivity parameter, and no significant interaction between condition and personality was found for either positive or negative viewing. Even when personality was examined, there were no differences between profiles, but rather between strategies. Indeed, for both the Resilient and Under-controlled profiles, acceptance failed to reduce negative expressivity and increase positive viewing, provoking more expressivity in both viewing. Conversely, suppression reduced negative expressivity, which is somewhat normal given its overt instruction to reduce expressivity. Conversely, acceptance produced more expressivity than suppression during negative and positive viewing. According to Dan-Glauser and Gross [38], this finding cannot be interpreted as simply inefficiency. Indeed, as the authors pointed out, greater expressivity may facilitate social interactions between people and, consequently, more successful and truthful communication.

In physiological parameters, our hypothesis of no significant difference from the reference value is confirmed for both acceptance and suppression, and we found a general lack of efficiency, except for HR in the Resilient group where acceptance presented to be efficient. When this group applied acceptance, it resulted in a softer decrease of HR during negative viewing, by itself and compared to suppression, indicating an efficiency of acceptance in HR. This is partially different from Dan-Glauser and Gross [38], who found over a short period of time a stronger HR decrease during suppression than during acceptance. In that case the analyses did not consider personality as an influencing factor in the efficiency of strategies. As far as the other parameters are concerned, i.e., SCL, RR, and RA, we think that, even if they usually display a fast reaction, it would be worthwhile to extend the trial duration to detect a potential efficiency produced by the strategies. Furthermore, these results imply an inefficient impact of acceptance and suppression, indicating that these strategies are probably not suitable to dampen the early physiological arousal, and perhaps antecedent-focused strategies like situation selection or distraction could be more appropriate, as the first analyses of an unpublished study performed in our research group indeed showed.

### Limitations and future directions

Considering that this study is one of the first attempts to define maladaptive profiles and assess the respective efficiency of ER strategies for these profiles, certain limitations must be taken into account, which provide suggestions for future studies.

First, in this study, we analyzed two personality profiles. This allowed us a deep focus on these groups and to compare the results with our prior study [156]. Nonetheless, as Rossi et al. [139] and Bastiaens et al. [17] reported, there are other maladaptive personality profiles that could be worth analyzing. For example, the “Over-controlled” or the “Anxious-Detached” profile, two maladaptive profiles found in previously published results, were not found in our sample. They differ in the intensity of the maladaptive traits within the profiles and, if tested, they could hypothetically lead to nuanced differences in the efficiency of ER strategies. Future research should investigate whether these additional profiles show distinct patterns of emotion regulation strategy efficiency. Second, although we tried to control this factor by asking participants at the end of the experiment if they correctly applied the strategy, it is possible that our participants, consciously or unconsciously, applied other strategies [72, 146] in addition to the one we instructed, or mixed the ones instructed. For instance, although the instructions were repeated at the beginning of each block of images, the instructions of the unregulated condition and acceptance may appear similar, potentially leading to overlapping regulated and unregulated responses during the experiment. In this experiment, the influence of context in the application of ER strategies was not further investigated. According to Aldao et al. [5], adequately choosing the best ER strategy according to the context could enhance the efficiency of strategies. Therefore, it is suggested to systematically consider the context in the analysis of ER efficiency in future studies. Third, some studies on acceptance suggested that a lack of training prior to the implementation of acceptance reduces its benefits [38, 136]. This may have occurred in the present study. It would be interesting to further investigate this strategy with prior training of participants. Fourth, as Wojnarowska et al. [168] pointed out, there is a potential confusion in the categorization of acceptance in the literature, which may lead to differences in the way it is instructed and, consequently, to greater variability between results. Indeed, some studies classify acceptance as an antecedent-focused strategy (e.g., [145, 164]), while others as a response-focused one (e.g., [38, 77]). In our study, we categorized acceptance as a response-focused strategy, implying a targeted regulation on the final emotional reaction. Nevertheless, a more consistent categorization in future literature would be necessary to avoid discrepant results between studies. Fifth, in this study, the stimuli lasted 8 s and therefore only allowed an assessment of short-term reactions. This restriction may have affected the physiological results, and it would be interesting in the future to test whether the use of longer stimuli could spot more differences in the emotional response dynamics. For instance, Gross and Levenson [69] investigated the efficiency of reappraisal and suppression in undergraduate students and they reported significant results in physiological parameters by using longer stimuli (between 1.5 and 4.5 min). Sixth, the reliability of some survey subscales is rather low. This may be related to the sample size and low onsistency in the responses. Future studies could control this by increasing the sample size and making it more heterogeneous. In this regard, this study tested a non-clinical population and, more specifically, undergraduate university students, which may have influenced the results through education biases. Hence, in future studies it would be interesting to include people from outside the university to extend this to the general population and avoid biases. Future research could benefit from including clinical populations to examine whether the patterns observed in our non-clinical sample replicate or diverge in clinical contexts, as suggested by findings from Pizarro-Campagna et al. [126] and Dixon-Gordon et al. [46]. A last limitation regarding participants concerns the exclusion criteria. At the beginning of the study, they needed to disclose if they had a diagnosis of mood or anxiety disorder, but this was not further verified by specific diagnostic interviews before the study. Considering the undetected diagnoses of these disorders among university students [30, 98], it could be relevant to examine the potential presence of psychological disorders in the studied population. Finally, given the potential psychometric flaws discussed in Sect. “Characterization of personality profiles: potential risk factors and emotion difficulties” about TAS and DERS, it would be interesting in future studies to consider other questionnaires to assess alexithymia and difficulties in ER, such as the Perth Alexithymia Questionnaire [128] and the Emotion Regulation Skills Questionnaire [63], the latter presenting nine subscales, thus allowing for a more comprehensive assessment of ER skills than the DERS.

## Conclusion

In this study, our aim was to focus on the maladaptive aspects of personality, by using the MPTM. Our sample clustered into Resilient and Under-controlled groups, which we can define as adaptive and maladaptive. Indeed, beyond obtaining high scores on maladaptive traits, this latter group was found to have more general ER difficulties, more negative affects, more difficulties in identifying feelings and a higher potential to suffer from anxiety and depression than the Resilient group. Interestingly, we found that Resilient people showed a stronger decrease of HR in emotional reactivity (unregulated), indicating a stronger attention orientation towards negative stimuli and, probably, that more resources are allocated to cope with negative stimuli in comparison to Under-controlled people. In the study of the efficiency of acceptance and suppression, we mainly found that, in experience, and regardless of personality profile, these strategies were not sufficiently efficient in adequately reducing experience during negative viewing over a short period of time. In this regard, other strategies like reappraisal [156] should perhaps be considered. Furthermore, acceptance was found to be efficient in decreasing the negative impact stimuli has on HR variation in the Resilient profile, indicating that acceptance could be suitable for people with higher psychological resources and fewer ER difficulties like Resilient people. For Under-controlled group, who reported higher ER difficulties, other strategies that prevent the development of unwanted emotions should be considered, such as situation selection [163]. Our study shows that suppression and acceptance have differential impacts depending on the parameters but that, overall, acceptance and suppression do not seem to be the best strategies to apply in these profiles, at least over a short period of time.

In conclusion, this study allowed us to gain a better understanding of maladaptive personalities, particularly in their psychological characterization. Knowing the characteristics of personality profiles regarding ER and their potential risks in the development of psychological disorders not only expand the knowledge in this field, but may also help clinicians to improve the understanding of the dysfunctional aspects of clients. Hence, it could allow to better shape treatments according to personality profiles. For instance, the Under-controlled group could benefit from the use of DBT [102], as it emphasizes ER skills-building and distress tolerance, i.e., aspects that are directly relevant to the difficulties observed in the Under-controlled group in our study. One could also turn to the BPD COMPASS model. The BPD COMPASS is a brief, personality-targeted intervention designed to reduce the maladaptive impact of traits within specific personality domains [141, 142]. Its modular structure allows clinicians to tailor treatment components to the individual’s predominant personality features [141, 142], making it a promising alternative to more generalized interventions such as DBT.

## Data Availability

The datasets used and/or analyzed during the current study are available from the corresponding author on reasonable request.

## Abbreviations

ER: Emotion Regulation
MPTM: Maladaptive Personality Trait Model
FFM: Five-Factor Model
EMG: Electromyography
ECG: Electrocardiography
HR: Heart Rate
SCL: Skin Conductance Level
RR: Respiration Rate
RA: Respiration Amplitude
DI: Difference Index
BPD: Borderline Personality Disorder
TAS: Toronto Alexithymia Scale
PANAS: Positive and Negative Affect Schedule
DERS: Difficulties in Emotion Regulation Scale
PHQ-4: Patient Health Questionnaire
PID-5-SF: Personality Inventory for DSM-5 Short Form
NEO-FFI: NEO-Five Factor Model
BEQ: Berkeley Expressivity Questionnaire
GAPED: Geneva Affective Picture Database
DBT: Dialectical Behavior Therapy
